# Organoid modeling reveals the tumorigenic potential of the alveolar progenitor cell state

**DOI:** 10.21203/rs.3.rs-2663901/v1

**Published:** 2023-03-14

**Authors:** Jingyun Li, Susanna M. Dang, Paul Schurmann, Antonella F.M. Dost, Aaron L. Moye, Margherita Paschini, Preetida J Bhetariya, Roderick Bronson, Shannan J. Ho Sui, Carla F. Kim

**Affiliations:** 1 Stem Cell Program, Division of Hematology/Oncology and Pulmonary & Respiratory Diseases, Children’s Hospital Boston, Boston MA 02115 USA; 2 Department of Genetics, Harvard Medical School, Boston, MA 02115, USA; 3 Department of Biology, Faculty of Science, Utrecht University, 3584 CH Utrecht, the Netherlands; 4 Harvard Chan Bioinformatics Core, Department of Biostatistics, Harvard T.H. Chan School of Public Health, Boston, MA 02115; 5 Rodent Histopathology Core, Harvard Medical School, Boston, MA 02115, USA; 6 Harvard Stem Cell Institute, Cambridge, MA 02138, USA

## Abstract

Alveolar type 2 (AT2) cells, the epithelial progenitor cells of the distal lung, are known to be the prominent cell of origin for lung adenocarcinoma. The regulatory programs that control chromatin and gene expression in AT2 cells during the early stages of tumor initiation are not well understood. Here, we dissected the response of AT2 cells to Kras activation and p53 loss (KP) using combined single cell RNA and ATAC sequencing in an established tumor organoid system. Multi-omic analysis showed that KP tumor organoid cells exhibit two major cellular states: one more closely resembling AT2 cells (SPC-high) and another with loss of AT2 identity (hereafter, Hmga2-high). These cell states are characterized by unique transcription factor (TF) networks, with SPC-high states associated with TFs known to regulate AT2 cell fate during development and homeostasis, and distinct TFs associated with the Hmga2-high state. CD44 was identified as a marker of the Hmga2-high state, and was used to separate organoid cultures for functional comparison of these two cell states. Organoid assays and orthotopic transplantation studies indicated that SPC-high cells have higher tumorigenic capacity in the lung microenvironment compared to Hmga2-high cells. These findings highlight the utility of understanding chromatin regulation in the early oncogenic versions of epithelial cells, which may reveal more effective means to intervene the progression of Kras-driven lung cancer.

## Introduction

Cell plasticity endows cancer cells the power to dynamically transition among different cell states, which can be defined by patterns of gene expression and chromation accessibility, without gaining additional genetic alterations. Cell plasticity has been implicated in cancer initiation, progression, tumor heterogeneity, and drug resistance ^1^. Targeting particular cellular states that contribute to cancer cell plasticity is held back by lack of facile in vitro models that recapitulate these states and limited understanding of the underlying mechanisms. Furthermore, it is not understood which cellular states are directly responsible for the functional properties of cancer cells, especially in early stages of disease. Answering these questions is particularly critical for improving lung cancer patient survival, since most lung cancers are diagnosed at advanced stages; thus, defining cell states present at early stages of lung cancer has promise to allow new methods to detect and intervene in advanced disease.

Mouse models, coupled with single cell (sc) sequencing (seq) techniques, have facilitated the study of cell plasticity and how they contribute to tumor heterogeneity in lung cancer. Kras activation (20-30%) and Trp53 loss of function (50-70%) are common mutational events in human non small cell lung cancer, and particularly in lung adenocarcinoma (LUAD), the most common type of lung cancer in patients ^2,3^. Activation of Kras alone (K) or together with P53 loss (KP) in GEMM are able to initiate clonal outgrowth of lung adenocarcinoma in vivo ^4^. Compared to the K model, the tumor progression in the KP model is more rapid with higher tumor burden, higher tumor grade and more metastatic disease. Single cell RNAseq and ATACseq indicated that alveolar type 2 (AT2) cells, which are the prominent origin cell type for lung adenocarcinoma in the Kras p53 mouse model, employ stereotyped programs during a time course of tumorigenesis ^5–8^. After the onset of Kras activation and P53 loss (KP hereafter), cells demonstrate gene expression similar to AT2 cells. Next, many other signatures including a mixed phenotype of AT2 and alveolar type I (AT1) cells (AT1/AT2 mixed) and a gastric-like signature are upregulated, indicating that AT2 cells are experiencing lineage infidelity in response to oncogenic Kras. At later stages, tumor cells exhibit the expression of genes related to mesenchyme and loss of epithelial state (EMT-like). Lineage tracing coupled with chromatin analysis of lung tumorigenesis in KP mice further indicated that AT2 cells follow two non-overlapping evolutionary paths for tumor progression, with a gastric-endoderm state in one path and mixed lung cell states featured in the second path ^9^. These findings indicate that AT2 cells have at least two sets of regulatory programs for tumorigenesis in vivo in the KP genetically engineered mouse model (GEMM).

Organoids are *in vitro* three-dimensional mini-tissues derived from normal progenitor cells or cancer stem cells. Unlike traditional *in vitro* models for tumor studies, including two-dimensional cancer cell lines and patient-derived xenograft models, tumor organoids derived from patient samples have been verified to recapitulate the histopathology, molecular profiles and response to therapies of their primary counterparts. Organoids derived from existing tumors, not from the normal cell of origin, have predominated the field. We recently established a tumor organoid system by co-culturing Kras activated (Kras or K hereafter) AT2 cells with lung mesenchymal cells (Dost et al. 2020), making it possible to characterize the molecular changes that occur in the transition of normal epithelial cells to tumorigenic cells. Using this system, we showed that Kras tumor organoids can robustly recapitulate the heterogeneity of primary tumors in GEMM and patient samples. After only seven days in culture, some cells in the Kras tumor organoids lost their original identity as AT2 cells, evidenced by decreased expression of AT2 cell marker genes and increased expression of lung developmental genes. We also observed heterogeneity, as some cells maintained AT2 signatures. However, in our Kras tumor organoids and in GEMM studies, it was unknown how the heterogeneous cell states related to key functions in tumorigenesis.

To investigate the possible functional differences between cellular states in LUAD and to define their regulatory mechanisms, we used single cell multi-omic sequencing to further characterize lung tumor organoids. Single cell multi-omic sequencing (10X) allows simultaneous profiling of gene expression and chromatin accessibility from one single cell, which provides a more precise understanding of mechanisms contributing to cell identity and state transition. We used KP tumor organoids to allow us to determine how this system models the early and late stages of LUAD in GEMM. Multi-omic dissection on KP tumor organoids indicated that seven day tumor organoids recapitulate the tumor heterogeneity of primary lung adenocarcinoma both at the gene expression level and the chromatin accessibility level. KP tumor organoid cells could be separated into two cell states resembling the states defined in GEMM, one resembling AT2 cells and one similar to the EMT-like state (Hmga2-high). We went on to use these findings to demonstrate that the AT2-like (SPC-high) state is important in tumorigenesis with further implications for plasticity in lung tumorigenesis.

## Results

### Single cell multi-omic profiling reveals two cell states exist in KP tumor organoids

We used single cell multi-omic RNA and ATAC sequencing to dissect the epigenetic and transcriptomic responses of AT2 cells to oncogenic events, including KrasG12D mutation and P53 loss. Firstly, AT2 cells were sorted from Kras^LSL-G12D^/P53^fl/fl^/LSL-YFP(KPY) GEMMs using well established surface markers (CD31−/CD45−/Epcam+/Sca1−)^10–14^. Kras activation and P53 loss were induced in the freshly sorted AT2 cells by infection with adenovirus 5 vector containing Cre recombinase driven by the ubiquitous cytomegalovirus (CMV) promoter (Ad5-CMV-Cre). Next, the Cre-induced AT2 cells were co-cultured with pre-expanded lung mesenchymal cells at an air-liquid interface for 7 days before harvest. We used 10X Genomics scMulti-omic sequencing to profile 7-days KPY tumor organoid cells, allowing gene expression and open chromatin profiling simultaneously from a single cell (Figure 1A). The gene expression UMAP and chromatin accessibility UMAP were generated separately using the scMulti-omic sequencing data (Figure S1A and S1B).

Next, we did cell clustering analysis using gene expression data and projected the cluster ID of each single cell onto the gene expression UMAP and chromatin accessibility UMAP (Figure S1A and S1B). We found that some clusters share a similar distribution on the chromatin accessibility UMAP even though they possess unique distributions on the gene expression UMAP (Figure S1A–C). We hypothesize that a cell state characterized by unique regulatory programs should possess unique signatures at both the gene expression layer and the chromatin accessibility layer. We merged the RNA clusters that share similar chromatin accessibility signatures to make sure that each cell state we defined not only possesses unique gene expression signatures but also possesses unique chromatin accessibility signatures. With this strategy, we defined four cell states to be Group 1 (Cluster 4), Group 2 (Cluster 2, 6, 7 and 9), Group 3 (Cluster 0, 1, 3 and 10) and Group 4 (Cluster 5 and 8) (Figure 1B–C and Figure S1C). Although we identified four distinct cell states, Group 1 shared many highly expressed genes with Group 2, and Group 3 shared many highly expressed genes with Group 4 (Figure S1D). Genes highly expressed in Group 1 and Group 2 were enriched for alveolar lamellar body and surfactant homeostasis Gene Ontology (GO) terms, while genes highly expressed in Group 3 and Group 4 enriched for in cell adhesion, cell junction and cell matrix adhesion, implying different cell function GO terms (Figure 1D–E). Consistent with our GO analysis, AT2 marker *Sftpc* and lung lineage specific transcription factor *Nkx2.1* show higher expression in Group 1 and Group 2. Group 3 and Group 4 show higher expression of *Hmga2*, which has been reported to regulate EMT programs during tumor progression ^7,11,15,16^ (Figure 1F). The patterns are also reminiscent of previous findings that lung adenocarcinoma has altered expression of lung lineage specifiers and oncogenic signal pathways ^17–20^. Our analyses suggest that there are mainly two distinct cell states that exist in the tumor organoids: an SPC-high state resembling AT2 cells and lung lineage specifiers (Group 1 and Group 2) and an Hmga2-high state with similarity to known oncogenic signaling pathways (Group 3 and Group 4). The two groups within each major state possess unique chromatin accessibility signatures, indicating more complex heterogeneity (Figure 1G–H). Herein we have largely focused on comparing the two major organoid cell states, SPC-high and Hmga2-high.

### Tumor organoids recapitulate the heterogeneity of *in vivo* tumors both at gene expression and chromatin accessibility level

Next, we wanted to know whether the cell states we observed in tumor organoids were reminiscent of the cell states from *in vivo* tumors. We compared our dataset with two studies focused on primary lung adenocarcinoma with the same genetic background (*KRAS*^*G12D*^/*P53*^*Loss*^). Marjanovic et al. did single cell RNA-seq of *in vivo* lung adenocarcinoma at seven stages, from pre-neoplastic to adenocarcinoma in the KP model ^6^. They defined 11 transcriptome programs to decipher the tumor heterogeneity during lung carcinogenesis. We checked the enrichment of those 11 transcriptome programs in the four cell states we identified in KP organoids and found that the two subtypes within each cell state tended to cluster together based on their enrichment of those 11 transcriptome programs (Figure S1E). The Hmga2-high organoid states are enriched for the EMT and highly mixed gene expression programs, with slight differences between Groups 3 and 4. Group 1 of the SPC-high tumor organoid state was highly enriched for the Marjanovic AT2-like and AT1/AT2 programs, whereas Group 2 was highly enriched for the gastric-like, stressed and GI epithelial-like programs. 11 chromatin co-accessibility modules were identified by LaFave et al., using scATAC-seq to characterize tumor heterogeneity at chromatin accessibility level from initiation stage to metastasis state in the KP model ^7^. The cell states we found in KPY tumor organoids also clustered together based on their enrichment of those 11 chromatin co-accessibility modules (Figure S1F). Within the SPC-high cells, Group 1 most enriched AT2-like, Nkx2.1 high, early gastric and AT1-like co-accessibility modules of LaFave et al, whereas Group 2 most enriched the late gastric and transition/Pre-EMT co-accessibility modules (Figure S1F). Within the organoid Hmga2-high cells, Group 3 most enriched the LaFave et al. NKX2.1 loss and late stage co-accessibility modules while Group 4 most enriched metastatic and RUNX2 high co-accessibility modules. These results indicated that the KPY organoids at a single time point can faithfully recapitulate the major cell states of *in vivo* KP lung adenocarcinoma both at the gene expression and at chromatin accessibility level.

### ScMulti-omic data dissects the regulatory programs underlying tumor heterogeneity

Since the organoid system can recapitulate the heterogeneity of in vivo tumor at the gene expression level and chromatin accessibility level, we used the multi-omic data to identify the regulatory programs underlying tumor cell states. For each transcription factor, we compared the expression level, motif enrichment score and target gene expression level between SPC-high cells and Hmga2-high cells. We identified the transcription factors for which all three layers of comparison were enriched in one specific cell state (Figure 2A–C). For example, we found that the expression level of Nkx2.1 is higher in SPC-high cells and the binding motif of Nkx2.1 is more enriched in the chromatin regions which are more open in SPC-high cells. The target genes of Nkx2.1 were also upregulated in SPC-high cells (Figure 2D–F). The result is consistent with previous findings that NKX2.1 expression helps to keep lung lineage identity and loss of NKX2.1 correlates with invasive phenotype^18,19,21^. Nfkb1 showed opposite patterns and is a candidate regulator for Hmga2-high cells (Figure S2A–C).

With our data analysis strategy, four key potential regulators were identified for SPC-high cells, including Cebpa, Foxa2, Nkx2-1 and Stat3 (Figure 2B and 2G–I). Previous studies have verified that Cebpa is gatekeepers for normal AT2 identity ^22,23^. Nkx2-1 and Foxa2 are co-expressed in normal AT2 cells and they can also coordinately regulate lung cancer cell growth and identity in a context-specific manner^18,19,21,24^. STAT3 has been confirmed to perform a tumor suppressive role in KRAS-driven lung tumorigenesis ^25,26^. STAT3 deletion can promote Kras driven tumor initiation and progression in mice ^26–28^. Those results indicate that the main transcriptome features of SPC-high cancer cells, which is lamellar body maintenance and surfactant hemostasis, may be guarded by the same regulators used by normal AT2 cells.

Six potential regulators were enriched in Hmga2-high cells, including Tcf12, Tcf4, Sox4, Rel, Nfkb1 and Hivep2 (Figure S2A–C and 2G–I). Tcf12 functions as a transcriptional suppressor for E-cadherin ^29^. Tcf4 has been reported to form complexes with β-Catenin to induce ZEB1, a key epithelial-to-mesenchymal transition activator^30–32^. Sox4 was found to function as a positive regulator of the β-Catenin signal pathway by upregulating the expression of Tcf4 ^31,32^. Rel and Nfkb1 are members of the NFκB transcriptional complex ^33^. Hivep2 are typical NFκB inhibitors ^34,35^, indicating that the NFκB signal pathway is dynamically regulated in the Hmga2-hi cells. NFκB signal pathway has been reported to evolve in tumor progression and metastasis ^36^. These findings here lend further support that the tumor organoid system can be used as an *in-vitro* system to identify and investigate the key regulators underlying cell states and tumor heterogeneity.

### SPC-high cells and Hmga2-high cells represent distinct path for tumorigenesis

Next, we used Monocle, which is an unsupervised approach, to calculate a tumorigenesis pseudotime trajectory across all cell states as a means to begin to understand the relationships between KPY tumor organoid states. The trajectory analysis revealed the trajectory as a fork, indicating two different tumor evolution paths (Figure 3A). Interestingly, the two groups of SPC-high cells mainly distributed on the left branch while the two groups of Hmga2-high cells mainly distributed on the right branch (Figure 3B–C). The expression patterns of Hmga2, Nkx2.1 and Sftpc were checked to verify the cell state distribution on the tumorigenesis trajectory (Figure 3D). The trajectory analysis suggests a gradual transcriptional transition within each state but the relationships between those two states are hard to infer since they are distributed on two branches. RNA velocity analysis suggests that there are limited transitions between SPC-high cells and Hmga2-high cells (Figure S3A–B).

To further define the cell states at the organoid level, we used immunofluorescence to detect markers of each state in KPY tumor organoid sections (Figure 3E). We used an antibody for Sftpc to identify the SPC-high cells and an Hmga2 antibody to label Hmga2-high cells. Three types of organoids were identified, including organoids only containing SPC+ cells (SPC+), organoids only containing Hmga2+ cells (Hmga2+) and organoids comprised of SPC+ cells and Hmga2+ cells (SPC+ Hmga2+) (Figure 3F). We found that about 89% of the organoids are either SPC+ or Hmga2+, suggesting that after oncogenic Kras activation, most AT2 cells give rise to one of those two cell states (Figure 3G). 7% of organoids possessed cells from both states (Figure 3G).

Next, we determined whether the cell states we identified in the KPY organoids also exist in the Kras^G12D/^LSL-YFP (KY) tumor organoids. We did scRNA-seq using 7 days of KY tumor organoids and then projected the cells onto the tumorigenesis trajectory of KP tumor organoids (Figure 3H). The density enrichment analysis of KY and KPY cells along the tumorigenesis trajectory indicate that K organoids tend to enrich SPC-high cells compared to KPY tumor organoids (Figure 3I). Interestingly, we found that the K tumor organoids are almost depleted of the Hmga2-high Group 4, raising the possibility that Kras^G12D^ mutation alone cannot drive AT2 cells to acquire the Hmga2-high Group 4 state, at least at the 7 day timepoint. This is consistent with previous findings that P53 loss can promote EMT-program activation ^37,38^. These results may suggest that tumors with different oncogenic genotypes may differ in cell states composition, providing further proof of concept for using the organoids system to dissect phenotypic variations in tumorigenesis.

### SPC-high cells have enhanced organoid forming ability in the presence of lung mesenchyme

We next sought to use cell surface markers to separate tumor organoid cells based on cell state features to facilitate comparison of the function of different cell states. Using differential gene analysis between the cell states, we found that CD44 can be used to distinguish SPC-high cells from Hmga2-high cells in 7 days KPY tumor organoids (Figure 4A–B). IF staining indicated that CD44-positive cells are Hmga2+ and Spc−, supporting the idea of using CD44 to subset tumor organoid cells (Figure 4C). Importantly, CD44 staining did not reveal distinct populations of cells in control organoids without Kras activation or from freshly isolated AT2 cells (Figure S4A–B). Next, we used FACS to subset cells from 7 days KPY organoids into CD44-High and CD44-Neg populations (Figure 4D). qPCR analysis indicated that CD44-High populations have higher expression of genes highly expressed in the Hmga2-high state, including CD44, Hmga2 and Slc4a11, whereas the CD44-Neg population enriched for cells with gene expression similar to the SPC-high state, including Sftpc, Cd36 and St3gal4 (Figure 4E). These results supported the rationale of using CD44 to subset and compare the KPY tumor organoid cell states.

Next, we used the organoid forming assay to investigate the organoid-forming potential of the two cell populations approximating the cell states. After subsetting 7 days KPY tumor organoids into CD44-High (enriched for Hmga2-high cells) and CD44-Neg (enriched for SPC-high cells) populations, we cultured those cells into organoids with (co-culture) or without (mono-culture) lung mesenchymal cells to compare the organoid forming efficiency (OFE) (Figure 4D) ; cells from established organoids were separated based on CD44 staining and replated into cultures either with or without mesenchymal cells. We found that both populations can grow tumor organoids in these conditions (Figure 4F–G). The OFE of CD44-high cells was not affected by the existence of mesenchymal cells, but the OFE of CD44-neg in mono-culture was significantly lower than that in co-culture with mesenchymal cells (Figure 4F–G). We also found that higher mesenchymal:tumor cell ratio correlates with higher percentage of SPC+ organoids, suggesting that mesenchymal cells may support the survival of induced AT2 cells that follow the SPC-high cell state (Figure S4C–D). To investigate whether those two cell states can maintain their identity after passaging, we quantified the types of organoids derived from the CD44-sorted populations in the two conditions (Figure 4H). Both CD44-neg and CD44-high cells could give rise to SPC+, Hmga2+, SPC+/Hmga2+ organoids, regardless if they are cultured with or without lung mesenchymal cells, indicating that there may be plasticity between the cell states (Figure 4H and Figure S4E–F).

### SPC-high cells exhibit higher tumorigenic capacity in vivo compared to Hmga2-high cells

Based on the results from the organoid assay, we hypothesized that SPC-high cell state has enhanced tumorigenic capacity in the lung microenvironment in vivo. We employed an orthotopic transplantation assay to quantify the tumorigenic capacity of the KPY organoid cell states ^10,11^. After sorting CD44-high and CD44-neg populations from 7 days KPY organoids, we transplanted the same number of cells from each population by intratracheal instillation (Figure 5A), and the mice were euthanized two months after transplantation to quantify the tumor burden. The mice that received CD44-neg cells had a significantly higher tumor burden than CD44-high cell recipient mice (Figure 5B and S5A). High grade tumors were observed in the recipient mice (Figure 5C). The CD44-low recipient mice had significantly more higher grade tumors (Grade III and IV) than CD44-high cell recipient mice (Figure 5C–D). We also performed immunofluorescence to infer the cell state potential of organoid cells after transplantation. We found CD44-high cell recipient mice had a higher percentage of Mixed tumors (SPC+/Hmga2+) whereas the CD44-low cell recipient mice had a higher percentage of SPC+ tumors (Figure 5E and S5B). Interestingly, the SPC staining intensity of most SPC+ tumor cells was comparable with adjacent normal AT2 cells (Figure S5C). Overall, the organoid and orthotopic assays indicated that the SPC-high cell state has a higher tumorigenic capacity in the lung microenvironment.

### Transcriptome signatures of cells state in tumor organoids can predict human LUAD survival rate

Since the KPY organoid cell states have different tumorigenic capacities in vivo and recapitulate the chromatin states in GEMM, we wanted to know whether they can be used to correlate with findings in patients. We first asked if similar cell states can be identified in human LUAD by comparing our dataset with a single cell RNA-seq data set from human LUAD published by Wang et al., 2021 (Figure S6A). They identified a group of cells (referred to as human LUAD AT2-like 2 cells) from both early and late stage patient samples, which closely resemble normal human AT2 cells. We checked the marker gene expression of each cell state from human LUAD to correlate to our organoid cell states. Our SPC-high cells (Group1) most resemble the human LUAD AT2-like 2 cells, whereas our Hmga2-high cells (Group4) most resemble the human LUAD AT2-like 3 cells in Wang et.al. We also used the top 10 highly expressed genes in each cell state to predict patient survival rate using publicly available data sets. We found that the genes highly expressed in the SPC-high state are associated with better survival rate (Log2(Hazard Ratio)<0) while the genes highly expressed in the Hmga2-high state are associated with poor survival rate (Log2(Hazard Ratio)>0) (Figure S6B–E). Group1 had higher power (*p*=0.0019) to predict a better survival rate than Group 2 (*p*=0.29). These results suggest that the chromatin-based cell states in murine tumor organoids may be useful to reveal key regulators of tumorigenesis in lung cancer patients.

## Discussion

Our murine tumor organoid system faithfully models lung cancer cells states at both the gene expression level and the chromatin accessibility level, providing a means to identify and test key regulators of tumor cell functions. By combining single cell multi-omic, pseudotime analysis, and organoid functional studies, our findings are consistent with the phylogenetic relationships proposed by others using genetically engineered mouse models, and further support that the organoid system recapitulates the common tumor evolution path of its in vivo counterpart. Yang et al. combined lineage tracing and transcriptome profiling to infer the phylogenetic relationship between cell states in the KP model ^9^. We further describe the changes in AT2 cells after the onset of Kras activation and p53 loss, in which AT2 can take one of two paths for tumorigenesis. The molecular and functional difference of cells on those two paths from our study are summarized in Figure 6A. Our results demonstrate many of the tumorigenic properties of the two cell states, particularly highlighting the capacity of SPC-high cell states, which most resemble AT2 cells, to drive tumorigenesis.

Our results, together with the established literature, support the idea of using organoid systems to dissect the distinct functional properties of cell states that contribute to tumor heterogeneity. Our previous study indicated that the tumor organoid system can model the early response of AT2 cells to Kras activation by comparing tumor organoids to control normal organoids ^11^. Here we more deeply characterized the KPY tumor organoids, particularly to show that the chromatin state of tumor organoids correlates with differences in tumorigenesis. Directly comparing the cell states we defined, KY tumor organoids have a higher percentage of SPC-high cells compared to KPY tumor organoids. KY tumor organoids did not exhibit evidence of the Hmga2-high Group 4 cell state. The induced lung adenocarcinoma in Kras^G12D^ mice grows much slower and shows lower metastasis frequency than tumors from Kras^G12D^/P53^Loss^ mice ^4^. It is intriguing to hypothesize that these differences in cell state composition may explain the difference in phenotypic characteristics between the K and KP models, such as metastatic potential. In organoid cultures, lung mesenchymal cells enhanced the growth of SPC-high cells, but did not impact Hmga2-high cells, consistent with the idea that the lung microenvironment supports the SPC-high cell state. Further investigation will be needed to determine if and how the cell states we and others observed contribute to metastasis. The chromatin accessibility and corresponding gene expression differences we found between the SPC-high and Hmga2-high states suggest that they may employ different regulatory programs for different aspects of tumorigenesis.

Our murine tumor organoid system and associated data provides a means to identify and probe the critical regulators of cell state and plasticity that may be driving human lung cancer. Our work shows that more emphasis is needed to understand and target the dependencies of AT2-like tumor cells, that is those cells that most closely resemble their normal counterparts. Many of the targets being pursued based on the literature and studies of advanced lung cancer are focused on the pathways we found are active in the tumor organoid Hmga2-high state, such as the Wnt and Nfkb pathways. Our study suggests that interrupting these signaling pathways may not target the AT2-like SPC-high cell state, which may be dependent on distinct pathways. Cells with the signature of our SPC-high cells were present in human lung cancer cells from very early stage samples; understanding this cell state may offer a means to interrupt the process of tumor progression in patients at earlier stages. Furthermore, SPC-high cells produced organoids with Hmga2-high cells after passaging in culture or transplantation in vivo and vice versa, suggestive of plasticity between the tumor organoid cell states. Thus, combination therapy targeting both cell states may be needed for more effective treatment of advanced lung cancer.

## Methods

### Mice

8-12 weeks old Kras^LSL-G12D/WT^; P53^flox/flox^; Rosa26^LSL-eYFP^ (KPY) mice was used to establish tumor organoids. 8-10 weeks old Athymic Nude mice were used for transplantation assay. All mice were maintained in virus-free conditions and all mouse works were approved by the BCH Animal Care and Use Committee, accredited by AAALAC, and were performed in accordance with relevant institutional and national guidelines and regulations.

### Isolating AT2 cells from mouse lung

Mouse lungs were dissected and digested for isolation of alveolar type 2 cells as previously described^14^. Briefly, mice were anesthetized with Avertin and fixed on the dissection platform. Expose mouse lungs and hearts. Perfuse the mouse lung with 10-15 ml PBS (ice cold) and intratracheal inject 2 mL dispase (Corning). Cut the lung out and minced into small pieces. Digest the lung pieces with 0.0025% DNase (Sigma-Aldrich) and 100 mg/mL collagenase/dispase (Roche) in PBS for 45 min at 37 °C. Gently vortex the cell mixture 2-3 times in the meantime. Filter the cell mixture sequentially with 100-μm and 40-μm cell strainers (Falcon). Centrifuge the cell mixture at 1000 rpm for 5 min at 4°C. Discard the supernatant and resuspend the cell mixture with red blood cell lysis buffer (0.15MNH4Cl, 10mMKHCO3, 0.1mMEDTA) for 90 s at room temperature. Quench the lysis with a 30ml PF10 buffer (10%FBS in PBS). Centrifuge the cell mixture at 1000 rpm for 5 min at 4°C. Resuspend the cell pellet with 1ml PF10 buffer. Four FACS antibodies, including anti-CD31-APC (Biolegend, 551262), anti-CD45-APC (Biolegend, 559864), anti-Ly-6A/E (SCA1)-APC/Cy7 (Biolegend, 560654), anti-CD326 (EpCAM) PE/Cy7 (Biolegend, 118216), were used at 1:100 concentration. DAPI (Sigma-Aldrich, 1:100) was used to eliminate dead cells. Single staining controls and fluorescence minus one (FMO) controls for all four channels were used to help decide the gate. FACS experiments were run on FACSAria II and FACS data were analyzed on FlowJo (BD).

### Induce AT2 cells and growing tumor organoids

To induce AT2 cells isolated from KPY mice, freshly sorted cells were counted and resuspended at 1 million cells/ml concentration in MTEC/Plus media^39^ containing 6X10^7^ PFU/ml of Ad5CMV-Cre. The cell and virus mixture were incubated at 37°C with 5% CO2 for 1 hour. Then wash the virus out with ice cold PBS. Cells were resuspended in 3D media (Dulbecco’s Modified Eagle’s Medium/F12 (Invitrogen) supplemented with 10% FBS, penicillin/streptomycin, 1 mM HEPES, and insulin/transferrin/selenium (Corning)) at a concentration to be 100,000cells/1ml. Neonatal lung mesenchymal cells were used as supporting cells as previously described^14^. The mesenchymal cells were suspended in growth factor reduced (GFR) Matrigel at a concentration of 1000,000cells/1ml. Mix the same volume of cell/3D media mixture and mesenchymal cell/Matrigel mixture and pipetted several times before adding 100μl mixture into a Transwell (Corning). Solidify the mixture for 20 min at 37°C, 5% CO2. Add 500μl 3D media into the bottom of the transwell. Change the media every other day.

### Dissociation of tumor organoids for FACS

Remove the 3D media in the bottom of the transwell and wash the bottom well with 500μl PBS once. 100ul warm dispase (Corning) were added on the top of transwells. Keep the plate at 37°C, 5% CO2 for 1 hour. Use 200μl tips to disrupt the matrigel structure and move all organoids mixture into a 1.5 ml falcon tube. Pellet the organoids with short spin and resuspend the organoids in TrypLE buffer for 5-10 min. Watch the cells every 3 min under microscope to make sure of single cell suspension. Quench the lysis prep with PF10 buffer. Anti-CD44-APC (Thermo Scientific, 17-0441-82) was used to subset different cell states in the tumor organoids. Single staining control and FMO controls were used for the FACS experiment.

### Single cell multi-omic sequencing

After digesting tumor organoids into single cells as described in “Dissociation of tumor organoids for FACS”, scMulti-omic sequencing was performed using the 10X genomics platform (Chromium Next GEM Single Cell Multiome ATAC + Gene Expression kit, PN- 1000285). Briefly, single cells from tumor organoids were used for nuclei isolation. Then the nuclei suspension was incubated with Transposition mix (10X Genomics) for DNA fragmentation in the open chromatin region. Then the transposed Nuclei were encapsulated on a 10X Genomics Chromium Controller with Chromium Next GEM Chip J. After pre-amplification, the RNA library and ATAC library are prepared separately. Quality control of RNA and ATAC libraries were run on the Agilent TapeStation High Sensitivity D5000 ScreenTape System, performed by Biopolymers Facility at Harvard Medical School. Libraries were sequenced by Bauer Core Facility of Harvard university using NovaSeq 6000 System.

### RNA extraction and quantitative RT-PCR

CD44-Neg and CD44-High population were sorted from 7 days tumor organoids as described in “Dissociation of tumor organoids for FACS”. Absolutely RNA Microprep Kit (Agilent) was used to extract RNA. Then complementary DNA (cDNA) was synthesized using the SuperScript III Kit (Invitrogen). RT-PCR was performed with TaqMan Assays and software as per the manufacturer’s recommendations.

### Orthotopic transplantation assay of tumor organoids

8-12 weeks Nude mice were used for transplantation assay. 1.5U/kg bleomycin were injected intratracheally 24 hours before the transplantation. CD44-Neg and CD44-High populations were sorted from 7 days KPY tumor organoids as described in “Dissociation of tumor organoids for FACS”. 50K CD44-Neg or CD44-High cells suspended in 45μl PBS were intratracheally administered into the bleomycin injured mouse lung. Recipient mice were sacrificed 2 months later after transplantation for histology evaluation.

### Organoid-forming Assay in Co-culture and Mono-culture condition

Digest primary KPY organoids (Passage 0) into single cells and subset the single cell suspension based on CD44 expression using FACS as described in “Dissociation of tumor organoids for FACS”. Then, CD44-High and CD44-Neg populations were plated again and grown into organoids in co-culture or mono-culture condition. For Co-cultured conditions, 2K CD44-Neg cells or CD44-High cells were suspended in 50μl 3D media and mixed with 50μl Matrigel containing 50k lung mesenchymal cells, then plate in one transwell. For Mono-culture condition, 2K CD44-Neg cells or CD44-High cells in 50μl 3D media and mixed with 50μl Matrigel, then plate in one transwell. Add 500μl 3D media to the bottom of transwells at both conditions and change the media every other day.

### Immunofluorescence and H&E

For tumor organoids staining, transwells were fixed with 10% neutral-buffered formalin overnight at room temperature, followed with dehydration by 70% ethanol overnight at room temperature. Carefully move Matrigel plug with organoids out of the transwell inset and immobilized with Histogel (Thermo Scientific). Send for paraffin embedding and cut into 5μm sections. Sections were rinsed in 100%, 95% and 70% xylene successively for deparaffinization, and then rehydrated with 100%, 95% and 70% ethanol. Next, sections were processed for hematoxylin and eosin (HE) staining or IF staining. H&E stainings were analyzed by at least two investigators including a pathologist with expertise in murine lung cancer (Curtis SJ, et.al. Cell Stem Cell. 2010; Rowbotham SP, et.al. Nat Commun. 2018). For IF staining, antigen retrieval was achieved by incubating organoids sections in citric acid buffer at 95°C for 20 min. Wash the sections with PBS-T (0.2% TritonX-100 diluted in PBS) three times and then block the sections with Block buffer (10% donkey serum in PBS-T) for 1 hour at room temperature. Several primary antibodies were used for IF staining, including CD44 (Thermo Scientific, 14-0441-82, 1:500), YFP (Abcam, ab6673, 1:400), SPC (Abcam, ab211326, 1:1,000), Nkx2-1 (Abcam, ab76013, 1:250), Hmga2 (GeneTex, GTX629478, 1:200). The primary antibodies were diluted in blocking buffer and then incubated with section overnight at 4°C. Wash the sections three times with PBS-T and incubated with secondary antibodies for 2 hours at room temperature. All secondary antibodies are from Invitrogen and used at 1:200 dilution, including anti-Rabbit Alexa 488, anti-Rabbit Alexa 594, anti-Mouse Alexa 594, anti-rat Alexa 647, anti-goat Alexa 488. Mount the slides with Prolong Gold with DAPI (Invitrogen).

### Single-cell multiome data processing and dimension reduction

Cellranger-arc (v 2.0.1) mkfastq was used to demultiplexes raw base call data to fastq files, and then cellranger-arc count program was used to map single-cell RNA and single-cell ATAC reads to the mm10 mouse reference genome. The cell-by-gene expression matrix was generated for the scRNA-seq data and the fragment files were generated for the scATAC-seq data as the outputs.

The scRNA-seq matrix was loaded into Seurat (v4.0, Ref: pmid 34062119) package in R for further quality control and processing. We applied the following filtrations at the per-cell level to keep the high-quality cells: number of gene > 200 & number of reads > 2500 & percent of mitochondrial reads < 10%. Cell cycle scores were regressed out during data scaling following the vignette of Seurat. For clustering, we took the first 2000 variable genes and performed PCA dimension reduction, followed by graph-based clustering and UMAP visualization using the top 60 principal components.

For the scATAC analysis, we utilized the ArchR package (v1.0, Ref: pmid 33633365) to read in the fragment files, and then performed quality controls by requiring the minimal number of fragments to be 1000 and the minimal TSS enrichment to be 4. Dimension reduction was carried out using iterative LSI (Latent Semantic Indexing) with the 500 bp tile matrix generated by ArchR. The parameters of iterative LSI were set as follows: iterations=3, resolution=0.2 and varFeatures= 50000. The ATAC cells were visualized using UMAP with the first 30 PCs from iterative LSI. Cell states annotation was based on both scRNA and scATAC signature as described in the main text.

### Integration of scRNA-seq data

Integration was carried out between the RNA data of KPY scMulti-omic profiling and the scRNA data of KY profiling. We firstly used SCTransform to perform count matrix normalization and identify the most variable genes, with the percentage of mitochondria regressed out. We then fed in the top 3000 most variable genes as anchors to perform integration between the two scRNA datasets. UMAP was used to visualize the integrated single-cell map.

### Differentially expressed genes and differential accessible peaks

Differentially expressed genes (DEGs) were identified with wilcoxon rank-sum test using the FindMarkers function of Seurat. The minimal percent of expression was set to 0.05, and the log fold change threshold was set to 0.5. Peak calling was carried out using MACS2 (v 2.1.1) on the pseudobulk of each cell state, with the adjusted p-value threshold set as 0.01. Differential accessible peaks (DAPs) were identified with wilcoxon test using the getMarkerFeatures function of ArchR, with TSS enrichment and number of fragments taken into account as confounding factors.

### Transcription factor analysis

Transcription factor (TF) analysis was performed with three different approaches, including single-cell chromVar-based enrichment, scenic-based regulon, and cell-state-specific TF expression. We first performed chromVar-based motif deviation analysis using the computeDeviations function of ArchR, with the genome-wide TF motif binding annotation from the cisbp database. We then used SCENIC package (v 1.3.1) to establish the TF-centric regulatory network, with cisTarget database used as TF target annotation. Lastly, we examined the expression of the TFs identified from the above two approaches. The TFs that are consistent across all three axes of motif enrichment, regulatory network and gene expression were defined as the TF regulators in each cell state.

### Trajectory analysis

We used the Monocle R package (v 2.22.0) to construct the trajectory of RNA cells at the per-cell level. The genes expressed in less than 10% of total cells were removed. The top 2000 differentially expressed genes between the SPC-high vs Hmga2-high were used to construct the pseudotime trajectory. The cell-cycle-related genes were removed from the differentially expressed gene list to minimize the effect from the cell cycle when construct the trajectory. In addition, we used the Velocyto (v 0.17.17) to constructing RNA velocity trajectory and expression dynamics, and then used the SeuratWrappers R package for visualization.

## Supplementary Material

1

## Figures and Tables

**Figure 1:** Two cell states exist in tumor organoids which are marked by unique gene expression signatures and chromatin accessibility signatures. A. Experimental pipeline of using single cell multi-omic sequencing to analyze KPY tumor organoids. AT2, alveolar type 2 (AT2) cells. TF, Transcription factor. B. Gene expression Umap showing the cell group identity. C. Chromatin accessibility Umap showing the cell group identity. D. Top 10 Gene ontology terms of genes highly expressed in Group 1 and Group 2. E. Top 10 Gene ontology terms of genes highly expressed in Group 3 and Group 4. F. Umap plots and boxplots highlighting expression level (Log(TPX+1), color bar of Umaps and Y axis of boxplots) of selected genes. G. Umap of gene expression assay showing the cell state definition in 7 days KPY organoids. H. Umap of chromatin accessibility assay showing the cell state definition in 7 days KPY organoids.

**Figure 2:** Using scMulti-omic data to dissect the regulatory programs underlying tumor heterogeneity A. Analysis strategy for identifying the transcription regulators underlying tumor heterogeneity. B. Venn diagram showing the overlap of TFs enriched in SPC-high cells using differential gene analysis, chromVar motif score analysis and Regulon expression analysis. C. Venn diagram showing the overlap of TFs enriched in Hmga2-high cells using differential gene analysis, chromVar motif score analysis and Regulon expression analysis. (D, E and F). Showing candidate regulator for SPC-high cells. The gene expression level (B), motif enrichment score (C) and Regulon expression score (D) of Nkx2.1 are shown both in boxplots and Umap. (G, H and I). Summary of all candidate regulators for SPC-high and Hmga2-high cells. The gene expression level (G), motif enrichment score (H) and regulon expression score (I) for each candidate regulator are shown using heatmaps. Regulators for SPC-high cells are colored blue. Regulators for Hmga2-high cells are colored red.

**Figure 3:** SPC-high cells and Hmga2-high cells represent distinct path for tumorigenesis A. Monocle 3 pseudotime trajectory analysis of scMulti-omic sequencing expression data of KPY organoids. B. Each cell state identified in Figure 1 G and H is illustrated in Monocle 3 pseudotime trajectory. C. Monocle 3 pseudotime trajectory analysis of gene expression assay of single cells from KPY organoids. Cells are colored by cell state identity. D. Gene expression level of selected genes along the pseudotime trajectory. Cells are colored by cell state identity. E. Experiment strategy for identifying SPC-high cells and Hmga2-high cells in 7 days KPY organoids using IF staining. (SPC was used to label SPC-high cells; Hmga2 was used to label Hmga2-high cells) F. Three types of KPY organoids, including Hmga2-high only (Hmga2+), SPC-high only (SPC+) and mixed (Hmga2+/SPC+) based on immunofluorescence staining of SPC and Hmga2. G. Quantification of the percentage of each type of organoids from F. H. Monocle 3 pseudotime trajectory analysis of integrated data (see method) containing scMulti-omic sequencing expression data of KPY (Kras^G12D^/P53^LOSS^/LSL-YFP)organoids and scRNA-seq data of KY (Kras^G12D^/LSL-YFP)organoids. Cells from KY organoids are colored Red. Cells from KPY organoids are colored by cell identity defined in Figure 1 G and H. I. Density distribution of cells from KPY or KY organoids along the pseudotime trajectory.

**Figure 4:** Co-culture with lung mesenchymal cells enhance the organoids forming ability of SPC-high cells but not Hmga2-high cells. (A and B) Gene expression level of CD44 on Umap (A) and in four cell states (B). C. IF staining indicates that CD44 co-stain with Hmga2. D. FACS strategy to subset 7 days KPY organoids using CD44, then the CD44-high and CD44-neg population were cultured into organoids in co-culture or mono-culture condition. E. qPCR showing that CD44 negative population expresses higher SPC-high lineage markers (Sftpc, Cd36 and St3gal5) and CD44 high population expresses higher Hmga2-high lineage markers (Hmga2, Cd44 and Slc4a11). F. Imaging showing transwells with organoids grown from SPC-high cells and Hmga2-high cells in Co-culture and Mono-culture conditions. G. Summary of organoids forming efficiency of SPC-high cells and Hmga2-high cells in co-culture and mono-culture conditions. Each dot indicates one biological replicate. *p-Value<0.05. N.s. non-significant. H. Quantification of the percentages of SPC+, Hmga2+ and mixed (Hmga2+/SPC+) organoids in passage 1 organoids derived from SPC-high (CD44-neg) and Hmga2-high (CD44-high) cells in co-culture and mono-culture conditions.

**Figure 5:** SPC-high cells have higher tumorigenic capacity than Hmga2-high cells in vivo. A. Experiment strategy for subsetting Hmga2-high cells and SPC-high cells and evaluating their ability of contributing to tumors in vivo using Orthotopic transplantation assay (see method). B. Quantification of tumor burden in CD44-High and CD44-Neg recipient mice. **p-value<0.001. C. HE staining shows representative tumors at different grades from CD44-Neg recipient mice and CD44-High recipient mice. D. Quantification of the percentage of tumor at different grades in CD44-Neg and CD44-High recipient mice. *p-value<0.05; E. Quantification of the percentage of each tumor type (SPC only, Hmga2 only and mixed) in CD44-Neg, and CD44-High recipient mice.

**Figure 6.** Model for oncogenic changes in AT2 cells after the onset of Kras activation and P53 Loss
