# Supplementary material for: Organoid modeling reveals the tumorigenic potential of the alveolar progenitor cell state
Source: Res Sq. 2023 Mar 14:rs.3.rs-2663901. Preprint. [Version 1] doi: 10.21203/rs.3.rs-2663901/v1 (PMC10055547; doi:10.21203/rs.3.rs-2663901/v1)
Supplement: 1 [file NIHPPrs2663901v1-supplement-1.pdf]

Supplementary Figure Legend

Figure S1, related to Figure 1. Cell state definition by combining gene expression assay and chromatin accessibility assay from one single cell.

A. UMAP projection of scMulti-omic sequencing expression data of KPY organoids. Cells are colored by RNA clusters.

B. UMAP projection of scMulti-omic sequencing chromatin accessibility data of KPY organoids. Cells are colored by cell clusters identified in **Figure S1A**.

C. Each cluster identified from scMulti-omic sequencing expression data (**FigureS1A)** is illustrated in Chromatin accessibility UMAP.

D. Heatmap showing the highly expressed genes in each group of cells.

E. Signature scores of gene programs (rows) (Marjanovic et al., 2020) in each cell state (columns).

F. Scores of chromatin co-accessibility modules (rows) (LaFave et al., 2020) in each cell state (columns).

Figure S2, related to Figure 2.

(A, B and C). Showing candidate regulator for Hmga2-high cells. The gene expression level (A), motif enrichment score (B) and regulon expression score (C) of Nfkb1 are

shown both in boxplots and Umap.

Figure S3, related to Figure 3. Pseudotime analysis reconstructs tumorigenesis Trajectory in tumor organoids.

A. RNA velocity analysis of 7 day KPY tumor organoids.

B. Cell states identity are plotted on the RNA velocity UMAP.

Figure S4, related to Figure 4. Co-culture with lung mesenchymal cells enhance the organoids forming ability of SPC-high cells but not Hmga2-high cells.

A. FACS strategy for subsetting two cell states from 7 days KPY organoids using CD44.

B. Check the expression of CD44 in freshly sorted AT2 cells (DAPI-/CD31-/CD45-/EPCAM+/SCA1-). CD44 FMO control was used to set the CD44-neg and CD44-high gate.

C. Representative pictures of whole mount staining on 7 days KPY organoids in different conditions when the ratio between epithelial cells and mesenchymal cells is 1:10, 1:5, 1:2.

D. Bar plot showing the percentage of SPC+ organoids in three conditions when the ratio between epithelial cells and mesenchymal cells is 1:10, 1:5, 1:2.

E. Representative pictures of 7 days SPC+/Hmga2- organoids (SPC-high), SPC-/Hmga2+ organoids (Hmga2-high) and SPC+/Hmga2+ organoids (Mixed) derived from CD44-neg population in Co-culture and Mono-culture condition. Scale bar, 100μm.

F. Representative pictures of 7 days SPC+/Hmga2- organoids (SPC-high), SPC-/Hmga2+ organoids (Hmga2-high) and SPC+/Hmga2+ organoids (Mixed) derived from CD44-high population in Co-culture and Mono-culture condition. Scale bar, 100μm.

Figure S5, related to Figure 5. SPC-high cells have higher tumorigenic capacity than Hmga2-high cells in vivo.

A. HE staining of lungs from PBS control, CD44-neg and CD44-high recipient mice.

B. IF staining showing the expression of SPC and Hmga2 in lesions from tumor organoids recipient mice. Both CD44-neg recipient mice and CD44-high recipient mice can derive SPC+, Hmga2+, SPC+/Hmga2+ tumors. Scaled bar=100μM.

C. IF staining showing the expression of YFP and SPC in lesions from tumor recipient mice. The expression of SPC in GFP+ tumor cells is comparable with adjacent normal AT2 cells. Scaled bar=100μM.

Figure S6, Transcriptional signatures of cell states identified in tumor organoids can be used to evaluate patient survival rate.

A. Umaps and boxplots indicating the signature gene expression of five human LUAD cell states identified by Wang et al., 2021.

B. Survival is enhanced (*p*-value=0.0019) in LUAD patients with higher expression of top 10 SPC-high Group 1 marker genes.

C. Survival is enhanced (*p*-value=0.29) in LUAD patients with higher expression of top 10 SPC-high Group 2 marker genes.

D. Survival is reduced (*p*-value=0.1) in LUAD patients with higher expression of top 10 Hmga2-high Group 3 marker genes.

E. Survival is reduced (*p*-value=0.046) in LUAD patients with higher expression of top 10 Hmga2-high Group 4 marker genes.
